# Bio-Based Sensors for Smart Food Packaging—Current Applications and Future Trends

**DOI:** 10.3390/s21062148

**Published:** 2021-03-18

**Authors:** Carolina Rodrigues, Victor Gomes Lauriano Souza, Isabel Coelhoso, Ana Luísa Fernando

**Affiliations:** 1 MEtRICs, Departamento de Ciências e Tecnologia da Biomassa, NOVA School of Science and Technology|FCT NOVA, Universidade NOVA de Lisboa, Campus de Caparica, 2829-516 Caparica, Portugal; victor.souza@inl.int; 2 INL, International Iberian Nanotechnology Laboratory, Av. Mestre José Veiga s/n, 4715-330 Braga, Portugal; 3 LAQV-REQUIMTE, Departamento de Química, NOVA School of Science and Technology|FCT NOVA, Universidade Nova de Lisboa, Campus de Caparica, 2829-516 Caparica, Portugal; imrc@fct.unl.pt

**Keywords:** active packaging, intelligent packaging, biopolymer, anthocyanins, pH sensor, food shelf-life

## Abstract

Intelligent food packaging is emerging as a novel technology, capable of monitoring the quality and safety of food during its shelf-life time. This technology makes use of indicators and sensors that are applied in the packaging and that detect changes in physiological variations of the foodstuffs (due to microbial and chemical degradation). These indicators usually provide information, e.g., on the degree of freshness of the product packed, through a color change, which is easily identified, either by the food distributor and the consumer. However, most of the indicators that are currently used are non-renewable and non-biodegradable synthetic materials. Because there is an imperative need to improve food packaging sustainability, choice of sensors should also reflect this requirement. Therefore, this work aims to revise the latest information on bio-based sensors, based on compounds obtained from natural extracts, that can, in association with biopolymers, act as intelligent or smart food packaging. Its application into several perishable foods is summarized. It is clear that bioactive extracts, e.g., anthocyanins, obtained from a variety of sources, including by-products of the food industry, present a substantial potential to act as bio-sensors. Yet, there are still some limitations that need to be surpassed before this technology reaches a mature commercial stage.

## 1. Introduction

Fresh food products are easily degraded due to physical injuries that could result from handling, transportation, storage, or by intrinsic factors caused by chemical reactions, enzyme action, and microbial spoilage [1,2]. In this sense, the use of packaging is a strategic choice for the protection and conservation of food [3] as it acts as a barrier to gases (oxygen, carbon dioxide), water vapor, and dust [4,5,6]. Therefore, packaging must be able to provide four main functions: protection, containment (small items can be grouped together in one package), information transmission (how to use, transport, recycle, or dispose of the package or product), and marketing (encourage potential buyers to purchase the product) [7,8]. It contributes to assuring the safety and quality of the food products for both suppliers and consumers, resulting in a reduction in food waste and more safety for the consumers [9,10]. Moreover, from an economical point of view, food packaging allows the manufacturers to avoid economical losses caused by the damages that can occur during transportation, management, and storage of the food; and the packaging also plays an important role in the marketing strategy of the product as it is the communication channel between the consumer and the industry [11,12,13].

Besides their benefits, packaging generates a huge waste and causes environmental problems since most of them are produced with non-renewable and non-biodegradable petroleum-based feedstocks. These materials have excellent properties, low cost and processability, however, they are not biodegradable, and they contaminate soil, air, and water, representing one of the 21st-century global issues [10,14,15]. Recently, new packaging materials are being studied as an alternative to the traditional petroleum-based ones. This is the case of biopolymers, that are obtained from renewable resources, and some with feasible functional properties, e.g., antimicrobial properties, such as chitosan [16,17].

Yet, packaging can also play an active role in the food preservation and may represent an information storefront on the state of the food being packaged [18], thus helping to fight food waste and food deterioration. In this regard, active and intelligent packaging are being studied and developed and represent an important mission in the food packaging industry.

Active packaging consists on the use of materials that can release, emit, absorb or scavenge substances directly or indirectly to the food to maintain quality or delay its degradation [19,20]. This packaging technology uses active compounds with e.g., antioxidant or antimicrobial properties that are incorporated into a polymeric matrix, coatings, or in labels, pads, or sachets [21]. The active compounds applied (such as phenolic compounds) can be obtained from, e.g., plants, which includes industrial crops such as kenaf, cardoon and hemp, algae, herbs, spices, fruits, or vegetables [22,23,24,25,26]. Active packaging can be divided into three main systems: antioxidant (e.g., butylated hydroxytoluene, vitamin C, vitamin E) [27], antimicrobial (e.g., essential oils, peptides, phenolic compounds) [28], scavengers or absorbers (e.g., oxygen scavengers, carbon dioxide absorbers or emitters, moisture control agents and ethylene absorbers or adsorbers) [29].

Intelligent packaging goes even further, by providing information to the consumer about the status of the packaged food using materials that monitor and interact with the packaging environment through an internal or external indicator [30,31]. Intelligent packaging can also be used to monitor the effectiveness of active packaging [1]. Intelligent packaging can be divided in three principal systems: indicators, sensors and data carriers [32].

Indicators determine the presence or absence of a substance, the extension of a reaction between different substances and the concentration of a specific substance. In general, indicators provide information through a visual change [33]. The majority of the indicators are focused on the detection of time-temperature, freshness, gas formation, and provides a color change that can be easily identified by the consumer [34].

Data carriers have the function to guarantee traceability, automatization, and to protect against theft and counterfeit. Data carriers are a way to transmit information within the food supply chain and do not have the function to contact with the product [32,35]. Data carriers most commonly used are barcode labels and Radio Frequency Identification (RFID) [32].

Sensors are electronic devices that are used to detect, locate or quantify an alteration through a receptor that is translated into a signal, using a transducer, that reports a physical or chemical change [33,36]. Biosensors are one type of sensors related to biological reactions, where a bioreceptor recognizes a biochemical sign and the transducer convert it into a quantifiable electronic response [19,35]. Biosensors can detect pathogens, allergens, and other toxic compounds [37].

Occasionally, in food packaging applications, the term “indicator” is often substituted by the term “sensor”. This result from the indicators “sensing” or monitoring action, expressed through a visual change, although indicators do not have the same features of sensors [38].

**Figure 1:**
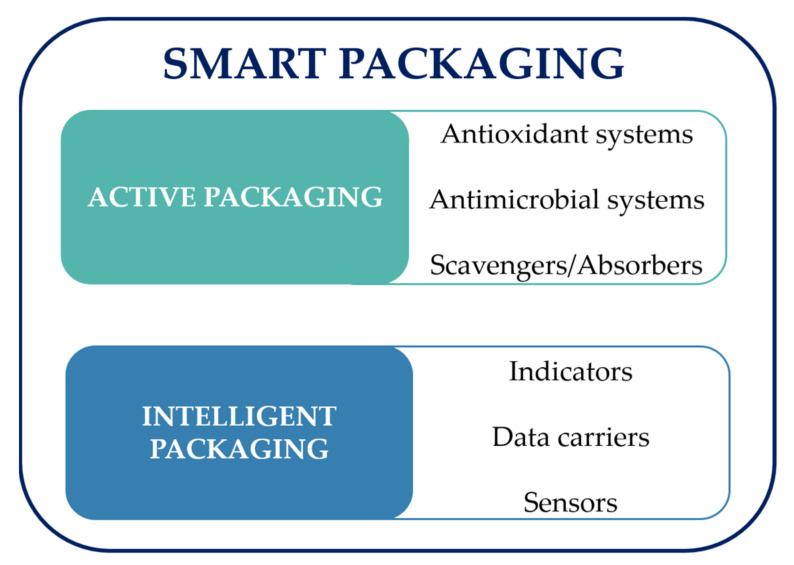
Schematic illustration of active, intelligent, and smart packaging.

Smart packaging comprises both active and intelligent packaging systems altogether, with the purpose to provide a more accurate information about the food product conditions to the consumer, and also display a protective effect over the food product through the use of, e.g., antioxidant and antimicrobial agents (Figure 1) [39,40,41]. Smart packaging can track a food product through the analysis and control of the internal or external environment, providing information to the entire food supply chain from manufacturers to consumers at any moment [42]. Despite intelligent and smart packaging being two distinct concepts, very often references to smart packaging are in fact to intelligent packaging [41].

As biobased materials have emerged as alternatives to the use of traditional synthetic plastics, their application as indicators and sensors is also arising. For example, natural dyes and extracts that may substitute synthetic ones with the ability to change their color depending on physiological variations of the food materials [3,43]. These pigments are generally incorporated into the polymeric matrix and change color, e.g., based on pH changes caused by microbial growth [18]. Thus, natural pigments can also constitute a more ecological and less toxic alternative to the already existing synthetic pigments that may cause harm to the consumers [44]. Besides, the use of biobased compounds as indicators or sensors is also in line with the current European Union policies that reinforce the need to increase the sustainability of the products by increasing their biodegradable and compostable character [45].

Therefore, this work aims to approach some of the most recent research developments on bio-based sensors to be used as intelligent packaging for food products. The use of natural compounds that can act as indicators may provide to the consumer an accurate response about the quality of the food and represents an alternative to the use of hazardous compounds. In addition, some of the bio-based sensors may also act simultaneously on the products being packaged (e.g., through its antioxidant activity), and therefore, the global packaging system represents a smart packaging. This review focuses on the applications and performance of biosensors when in contact with perishable food products. Critical analysis of the most recent information available will be useful to identify future opportunities for the use of bio-based sensors in food packaging systems, helping to contribute to the development of more sustainable packaging.

## 2. Developments in Bio-Based Sensors

Developments on bio-based sensors (i.e., sensors that are using compounds obtained from natural products and using biopolymers) to be used as intelligent packaging for food products, are mainly focusing on freshness indicators.

Indicators have the function to provide information about changes occurring in a food product or in the surrounding environment, providing more accurate information about the status of a food product for the consumers [36].

Freshness indicators are used in intelligent packaging to provide information about the quality decay of the food [34]. These type of indicators are mostly based on a visual colorimetric response, caused by a chemical or microbiological change of the food product that reacts with the sensor indicating changes in the product, generally caused by a pH alteration [46]. The freshness sensor or indicator can be applied in the form of a label, tag, or visual indicator [38]. Freshness indicators can be divided into direct or indirect indicators. Direct indicators are the ones which action is based on the direct reaction with a specific metabolite or toxin produced, whereas the indirect ones are based on an indirect reaction caused by chemical or microbiological changes that modify the environment where the food is placed [47,48].

To perform their function and help in the maintenance of food quality, freshness indicators are based on a color change of pigments that are added to the indicator and are reactive to pH changes. Synthetic dyes are commonly used such as methyl red, bromocresol purple, bromophenol blue, chlorophenol red have been proved to be able to react to pH changes when integrated into intelligent packaging systems [34,49]. Although they are demonstrated to be effective, they are not fitted to be used for food applications due to their toxic, carcinogenic, and mutagenic characteristics that threaten not only the consumers’ health but also the environment [50].

The use of natural dyes instead of synthetic ones fits the consumption trend for sustainable and more natural healthy choices [49]. Natural pigments extracted from plants and fruits are renewable, demonstrate low toxicity, and low environmental impacts [50]. To be used as a pH indicator it is mandatory to consider the stability and the pH response of the pigments, which depends on each type [3,51].

Recently, the use of natural color pigments in biopolymeric matrices in food packaging systems has emerged, fitting this trend of the awareness for sustainable choices [3]. Additionally, the majority of the natural pigments and biopolymeric matrices possesses bioactive properties not only acting as a pH indicator but also by providing active properties to the indicator and for the food product [33,52].

## 3. Bio-Based Sensors—Applications

Microbial growth in food is a cause of food degradation leading to a loss of freshness by producing metabolites that can be detected by indicators present on the packaging. The presence or the formation of metabolites such as volatile nitrogen compounds, carbon dioxide, biogenic amines, ethanol, or sulfurous compounds are examples that can be detected through freshness indicators [33,53]. Meat, fish, seafood, fruits, and vegetables are perishable products and assuring their quality is very important to protect the consumer from hazardous metabolites produced during its degradation process. Meat freshness decreases with microbial growth leading to the formation of several compounds like acetic acid, n-butyrate, and biogenic amines [47]. The formation of these compounds compromises meat quality and so the freshness indicator can be used to detect them. In fish and seafood, spoilage is mainly indicated by the amount of total volatile basic nitrogen (TVB-N) and the presence of biogenic amines resulting from the decarboxylation of amino acids due to the microbiological action [54]. Additionally, the production of vapors or gases such as ethanol or carbon dioxide (CO_2_) produced by the action of microorganisms decays food quality and the metabolites can be detected by the freshness sensors [55].

Several studies have been arisen through the past few years concerning the development, evaluation, and application of biobased sensors using natural compounds in intelligent food packaging and some of them are reported and summarized in Table 1. In these studies, examples focused mainly on bio-sensors associated with biopolymers, but also some other synthetic polymers that are easily degraded were also added as examples. In the table, it was also indicated which was the action and function of the sensor and how they respond to the loss of freshness in the food. Most of the sensors are sensitive to a pH alteration caused by the release of volatile nitrogen compounds, and this change is consequently identified by a colorimetric response. Sensors are generally placed into the headspace of the food container, avoiding direct contact with food but close enough to detect media alterations and providing a response to food quality alterations. When these biosensors are associated with the biopolymers, they are usually incorporated in the polymeric structure and the modification in the color of the films indicates the food quality alterations of the product being packed. The information collected also clearly shows that extracts rich in pigmented chemical compounds, that change color with pH, have been intensively studied and used in these bio-sensors, especially anthocyanins. Additionally, most of the studies applied the bio-sensor in fish, meat, and seafood, most probably because their quality decay represent an important economic loss and also because, in the degradation process, the pH of the surrounding environment changes, this change being easily detected through the use of those pH-sensitive-biosensors. Interestingly, some of the compounds applied and tested in the sensor, not only present a pH-sensitive color but also have other bioactive properties, e.g., antimicrobial properties, and its presence in the polymeric matrix can also enhance the preservation activity of the packaging material. Yet, the incorporation of those compounds in the biopolymer can also change some of the mechanical, barrier and optical properties of the films [56,57]. Consequently, in some applications, it might be necessary to reach a balance between the beneficial action of the pH bio-sensor activity and its adverse effects on some of the properties of pristine biopolymers (e.g., mechanical properties). Following the information collected and summarized in Table 1, a wider description will be completed to clarify the significance of the studies.

**Table 1:** Food application of bio-based pH sensors.

Matrix	Sensor Material (Source/Type)	Function	Major Results	Food Application	Ref.
Gelatin-Gellan gum bilayer films	Mulberry fruits—anthocyanins	Sensitivity of the films to volatile nitrogen compounds through pH change	The use of ZnO nanoparticles improved the color stability of the films; Bilayer films proved to be sensitive to ammonia; Gellan gum-Mulberry anthocyanins/Gellatin 2.0% film demonstrated visible color changes linked with crucian spoilage	Crucian fish	[58]
Starch	Grape skin—anthocyanins	Sensitivity of the films to volatile nitrogen compounds through pH change	Anthocyanins caused changes in the mechanical properties; Cassava starch sheets with the highest content in anthocyanins proved to be effective to act as a pH-sensor, showing color changes when the pH environment changed.	Beef and fish	[59]
Agarose	Red cabbage and rose—anthocyanins	Sensitivity of the films to volatile nitrogen compounds through pH change	The indicator sensor is reactive to an increase in ammonia production, causing conformational changes on anthocyanins and a color change was observed; Under simulating conditions of ammonia production, the indicator turned from red to green; Same results were observed when the indicator was in contact with buffalo meat.	Buffalo meat	[60]
Chitin nanofiber and methylcellulose	Red barberry—anthocyanins	Sensitivity of the films to volatile nitrogen compounds through pH change	Mechanical, optical, and physical-chemical properties of the films changed when anthocyanins were added to the chitin nanofiber/methylcellulose film; A colorimetric pH-sensitive response was observed when the films were placed in contact with spoiled fish in containers.	Fish	[61]
Pectin	Red cabbage—anthocyanins	Sensitivity of the films to volatile nitrogen compounds through pH change	A color change (purple to yellow) was observed when exposed to different amines; Same behavior was observed when the film was introduced in the headspace of the package containing spoiled food products.	Beef, chicken, shrimp and fish	[62]
Chitosan, carboxymethyl cellulose	Blueberry and red grape skin pulp—anthocyanins	Sensitivity of the films to volatile nitrogen compounds through pH change	Extracts did not affect mechanical properties of the films produced; Color changes occur in both films; Color changes were more evident on carboxymethyl cellulose than on chitosan films.	Chicken meat	[50]
Carboxymethyl cellulose sodium and *Artemisia sphaerocephala* Krasch. gum	Red cabbage—anthocyanins	pH and NH_3_	Red cabbage extract influenced mechanical and optical properties; Films changed from rose-bengal to aquamarine when the pH of buffer solutions went from acidic to alkaline conditions. A more evident color was observed when in contact with NH_3_ rich-atmosphere.	Not applied	[63]
κ—carrageenan	*Lycium ruthenicum* Murr. —anthocyanins	Sensitivity of the films to volatile nitrogen compounds and to lactic acid production through pH change	Films in contact with milk produced a color change from grayish purple to dark pink due to lactic acid production; (acidic conditions); Films when exposed to shrimp caused a color change from grayish purple to yellow due to volatile amines production; (alkaline conditions).	Milk and Shrimp	[64]
Starch and gelatin	Red raddish—anthocyanins	Sensitivity of the films to volatile nitrogen compounds through pH change	A color response was observed in a pH range from 2 to 12; As a response to volatile nitrogenous compounds produced from prawn and poultry meat spoilage, films changed from red to grey-purple.	Prawn and poultry meat	[65]
Methylcellulose	Jambolan fruit—anthocyanins	pH	A color response was observed in a pH range from 1–10; Films changed their color from red/pink under acidic conditions to purple/blue under alkaline conditions.	Not applied	[66]
Chitosan and TiO_2_	Apple pomace—anthocyanins	Sensitivity of the films to volatile nitrogen compounds through pH change	Films changed their color from red to light pink when the salmon was deteriorating.	Salmon	[67]
Composite of polyvinyl alcohol, microcrystalline cellulose and polyvinyl pyrrolidone	Red cabbage—anthocyanins	Sensitivity of the films to volatile nitrogen compounds through pH change	Foams with anthocyanins were successfully developed; Foams changed their color when a pH change occurred; Foams changed from purple to greenish-grey after 3 days in contact with prawn and chicken meat, aligned with food products degradation.	Prawn and chicken	[68]
Chitosan and Oxidized chitin nanocrystals	Black rice—anthocyanins	Sensitivity of the films to volatile nitrogen compounds through pH change	Films reacted to the contact with shrimp and promfet, and a visible change of color was observed; Films changed from purple to grey-blue color aligned with the food degradation	Shrimp and promfet	[69]
Quaternary ammonium Chitosan-Polyvinyl alcohol	Cactus pears extract (CPE)—betalains	Sensitivity of the films to volatile nitrogen compounds through pH change	Color of the films changed when CPE was added; CPE addition affected mechanical, structural, antioxidant and antimicrobial properties of the films; Films showed sensitivity to volatile nitrogen compounds present on the shrimp container.	Shrimp	[70]
Quaternary ammonium chitosan—Fish gelatin	Amaranth—betalains	Sensitivity of the films to volatile nitrogen compounds through pH change	Physical and functional properties of the films were enhanced by amaranth extracts; pH changes due to the increase in volatile nitrogenous compounds caused a color change from pink to yellow.	Shrimp	[71]
Starch-Polyvinyl alcohol	Red pitaya—betalains	Sensitivity of the films to volatile nitrogen compounds through pH change	Physical and functional properties of the films were enhanced by red pitaya extracts; pH changes due to the increase in volatile nitrogenous compounds caused a color change from pink to yellow.	Shrimp	[72]
Gelatin-Polyvinyl alcohol	Amaranth—betalains	Sensitivity of the films to volatile nitrogen compounds through pH change	Physical and functional properties of the films were enhanced by Amaranth leaf extracts; Films changed color from red to yellow following food products degradation.	Chicken and Fish	[73]
Cellulose	*Arnebia euchroma*- Naphthoquinone	Sensitivity of the films to volatile nitrogen compounds through pH change	Films demonstrated a color response in a pH range from 5 to 12; Films changed their color from rose-red to blue-violet when pH changes occurred.	Pork and shrimp	[74]
Cellulose paper	Gromwell (*Lithospermum erythrorhizon*)—Shikonin	Sensitivity of the films to volatile nitrogen compounds through pH change	Films changed from red to blue because of fish and pork degradation.	Fish and Pork	[75]
Agar—Polyvinyl alcohol	Curcumin	Sensitivity of the films to volatile nitrogen compounds through pH change	Films changed their color from, yellow to orange-red due to ammonia production from shrimp degradation.	Shrimp	[76]
Tara gum and Polyvinyl alcohol	Curcumin	Sensitivity of the films to volatile nitrogen compounds through pH change	Films changed their color from, yellow to orange-red due to ammonia production from shrimp degradation.	Shrimp	[77]
Pectin and sulfur nanoparticles	Curcumin	Sensitivity of the films to volatile nitrogen compounds through pH change	Addition of curcumin and sulfur nanoparticles improve thermal stability and UV light barrier. A color change from yellow to orange was observed caused by shrimp degradation.	Shrimp	[78]
*Lallemantia iberica* seed gum	Curcumin	Sensitivity of the films to volatile nitrogen compounds through pH change	Mechanical and barrier properties of the films were enhanced by Amaranth leaf extracts; Films changed their color from, yellow to red due to ammonia production from shrimp degradation.	Shrimp	[79]

### 3.1. Anthocyanins Used as Bio-Based Sensors

**Figure 2:**
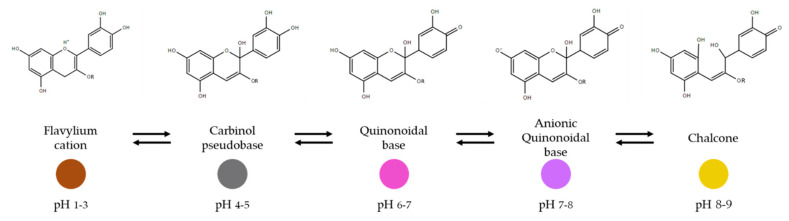
Changes in anthocyanins structure due to pH changes, adapted from [18].

Anthocyanins are natural water-soluble pigments present in several plants and fruits, they are classified as flavonoids and their antioxidant, anti-inflammatory and anticarcinogenic properties are reported in the literature [18]. They are responsible for red, purple and blue color of a variety of plants and fruits and highly reactive to pH changes (their chemical structure changes with the changes in the pH, which result in different colors) [51,80] (Figure 2).

A pH-sensitive bilayer hydrogel film was developed using one layer made of gelatin and ZnO nanoparticles incorporated and the second layer made of gellan gum with anthocyanin extracts to monitor fish spoilage [58]. Anthocyanins were extracted from mulberry fruits and their response to pH variations was studied. From pH 2 to 6, they show colors going from light pink to colorless. From pH 7 to 12, anthocyanins change their color from light yellow-green to orange color. The color stability of anthocyanins was improved by using ZnO nanoparticles on the upper layer of the film, acting as a UV-light barrier. Additionally, the bilayer film is suited for electrochemical writing. To monitor fish spoilage, crucian was placed in a box with a lid and a hole was made on it, and the bilayer film was placed covering the hole. Total volatile basic nitrogen (TVB-N) content and color changes were evaluated, showing that after 6 days refrigerated storage, the color changes occurred on the film, going from an initial pink to light green, and finally yellow as a result of the increase in the TVB-N content, which represents the degradation of the fish packaged. The color changes on the film are linked with the production of NH_3_, which could hydrolyze the hydroxyl group (OH-) present on the hydrogel film and create alkaline conditions. Under these conditions, anthocyanins changed their structure from flavylium ion to a chalcone structure that is noticed by film color changes [51]. The use of nanoparticles improved the stability of the films and did not interfere with NH_3_-sensing properties. Additionally, the electrochemical writing ability of the films constitutes a green printing technique that allows the display of the information directly on the film, constituting an innovative way to present important information for the consumers. The films developed can act as an intelligent sensor on the detection of fish spoilage [58]. However, no tests were done regarding their antimicrobial and antioxidant activity.

The use of anthocyanins as a pH sensor to monitor beef and fish was studied by Vedove and collaborators [59]. Cassava starch sheets were produced by extrusion process with the introduction of anthocyanins during the process. Cassava starch sheets with different concentrations of anthocyanins (5, 10, and 20 mg per 100 g) were then placed on top of a falcon tube and samples of beef and fish were introduced in the tube, kept at 6 °C (beef) and 22 °C (fish), and the changes were evaluated for 3 days. The mechanical and physical properties of the cassava starch sheets were altered by the introduction of anthocyanins, resulting in an increase in swelling, reduction in flexibility and lower hydrophilicity compared to pristine cassava starch samples. As food degradation started, gas was produced (due to TVB-N production), pH increased, and the headspace environment became more alkaline. The gas production causes an hydrolyzation of the anthocyanins present on the cassava starch sheets and consequently, a slight color change occurred. The color change was more intense on the cassava starch sheets with a higher content of anthocyanins on both food products, nevertheless, authors reported that an improvement in the production and formulation is needed since a poor color change can confuse the consumers [59]. No tests were performed in order to evaluate the antimicrobial and antioxidant properties of the films. Anthocyanins extracted from red cabbage and roses were also studied to act as a pH indicator sensor to evaluate meat food spoilage [60]. Anthocyanins were mixed in an agarose solution and left to dry, and the performance of the indicator was evaluated firstly in vitro tests under ammonia production that simulates the meat degradation process, and then in situ in contact with buffalo meat. Once again, a color change occurs, going from red to green when the pH increases, a consequence of the production of volatile nitrogenous compounds that causes a conformational change of the structure of the anthocyanins [81]. Both tests demonstrated the potential of the film to be used as freshness indicator. However, no tests to access the antimicrobial and antioxidant activity were done. Other authors developed pH-sensitive films based on chitin nanofibers and methylcellulose with anthocyanins extracted from barberry (*Berberis vulgaris* L.) fruits as sensing material [61]. Films were produced by casting method and the anthocyanins extract was introduced in the chitin nanofiber and methylcellulose film solution. To assess the pH-sensitive films capacity and their response under high levels of ammonia, films were introduced on the fish packaging container and evaluated for 72 h. Once again, when the pH environment changed due to ammonia production, a color change from reddish to pale pink was observed indicating food degradation. However, these pH-sensitive films, were tested not only in terms of colorimetric response to the pH change, when facing an ammonia content increase, but also in terms of potential antibacterial and antioxidant activities. It is noteworthy that this is an important feature of smart packaging: the combination of intelligent properties conferred by the color changes provided by anthocyanins as a reaction to food degradation with active properties being important for food preservation. Indeed, films demonstrated to have a strong antioxidant capacity similar to a synthetic antioxidant (BHT). Nevertheless, this latter feature was only accessed on films and not on food products. Moreover, the effect of anthocyanins on the film matrix appeared to improve mechanical, optical, and physical-chemical properties.

Concerning nitrogenous volatile compounds, a sensor was developed consisting of a film of pectin with red cabbage extract rich in anthocyanins that were incorporated as an indicator [62]. The sensor was produced through casting method and the red cabbage extract was added to the filmogenic dispersion before it was left to dry. The use of plasticizers was determinant to maintain the integrity of the films. The effectiveness of the sensor was firstly tested by exposing the film to several amines and verified through UV-Vis spectroscopy, as amines causes a pH change to which anthocyanins are highly reactive changing films color from purple to orange or yellow [5]. Tests on different food products (beef, chicken shrimps and fish) were assessed by attaching the film to the headspace of the different food products containers. It was demonstrated a similar color change from purple to yellow that fits the results observed for the volatile nitrogenous compounds production and the increase in microbial growth. Films were made of edible components and demonstrated their potential to be used as freshness indicators in intelligent packaging. However, no tests were done regarding their antimicrobial and antioxidant activity.

Blueberry and red grape skin extracts were applied in two different matrices (chitosan and carboxymethyl cellulose) to monitor poultry meat freshness [50]. The addition of the extracts to the polymeric matrixes did not affect the mechanical properties of the films. Due to the different constitution of the polymeric matrixes, the use of anthocyanins rich extracts caused different color changes when in contact with poultry meat. The films were placed directly on the poultry meat and when the food started to deteriorate a color change on the films was observed affected by a change in the pH caused by the production of amines, turning into alkaline conditions. Carboxymethyl cellulose (CMC) films changed their color from violet to pink when blueberry extract was used and from red to pink for red grape seed extract. In chitosan films, the color changes were less evident due to the difference between polymeric structures and rearrangements after the addition of anthocyanins, making the first polymer matrix more suitable to incorporate the extract and to be used as a pH sensor in poultry meat. In these tests, films were only essayed for its intelligent character and not for its active character.

*Artemisia sphaerocephala* Krasch. gum was used as a film-forming solution with the addition of carboxymethyl cellulose sodium as host-complex for red cabbage extract rich in anthocyanins [63]. Thus, a film was prepared to act as a pH and NH_3_ indicator. To improve anthocyanins stability (positively charged), carboxymethyl cellulose was added due to its attraction to positive and negative charges, avoiding anthocyanins migration [82]. Red cabbage extract influenced the mechanical and optical properties of the films. In contact with different buffer solutions, the color of the films changed from rose-bengal to aquamarine color and proved to be effective to act as a sensor for pH changes. Furthermore, NH_3_ response was observed by a more pronounced color change. In this study, antimicrobial and antioxidant activities were not accessed.

Milk and shrimp freshness were evaluated through a pH sensor indicator film made of κ—carrageenan and *Lycium ruthenicum* Murr. extract rich in anthocyanins [64]. Different concentrations of extract (1.5, 2.5, 3.5, 4.5 *wt*%) were used and it was noted that an increase in extract concentration affected the film structure, optical, mechanical, and barrier properties negatively, so only the film with 2.5 *wt*% of the extract was used to monitor milk and shrimp freshness. A color change from grey to dark pink was observed when the film was in indirect contact with milk after 48 h (the film was placed on the top of the milk container). The color change occurred due to a decrease in the pH of the milk, caused by the production of lactic acid. On the other hand, a change in the pH from acidic to alkaline conditions caused by volatile amines produced by the shrimps turned the films from light grey to yellow in 72 h [64]. Lower concentrations of extract end up being more suitable to be used to produce films, with fewer effects on the film structure. Besides the colorimetric response to pH changes, the films also demonstrated good antioxidant activity turning them suitable to be used in smart packaging. However, in this study the active action of the packaging directly on food was not addressed.

Anthocyanins from red radish were used to develop a pH-sensitive film made of starch and gelatin [65]. The anthocyanins rich extract was introduced on the film-forming dispersion after the gelatin and starch and cast. Films showed a color response in a pH range from 2 to 12 when exposed to different solutions and demonstrated good mechanical properties. Films were attached to the top of Petri dishes to have contact with poultry meat and prawn environment during storage (0 °C, 4 °C, and 30 °C). At any temperature and for both food products, films changed their color from red to grey–purple, because of the production of volatile nitrogenous compounds that caused deprotonation of anthocyanins [49]. The use of two biopolymers that interact with the anthocyanins avoided the pigment to be released into the water. Storage temperature was an important factor for anthocyanins stability once there is a preference for lower temperatures [83]. The antimicrobial and antioxidant activities were not evaluated in the study.

Jambolan skin extract is also a source of anthocyanins, was used as a sensing material to develop pH reactive films [66]. Jambolan is a fruit with low economic value and is highly perishable so the valorization of the skins for the recovery of anthocyanins for packaging use adds value for this particular product [84]. The extracts were incorporated into the film-forming dispersion in different concentrations (10, 30, and 50% *v*/*v*), using methylcellulose as a matrix to develop films through the casting method. When exposed to different buffer solutions (pH range 1 to 10), all films showed a colorimetric response, changing their color from red and pink (acidic conditions) to purple and blue (alkaline conditions). Jambolan fruit extracts improved the mechanical and barrier properties of the films and demonstrated an increase in antioxidant activity [66]. Therefore, the films proved to be well suited to smart packaging due to their ability to provide a colorimetric response and also to demonstrate antioxidant ability, both by the action of anthocyanins. Further studies are needed to address the active action as a packaging material since it was only studied on standalone films.

Red apple pomace extract was incorporated into chitosan-based films with TiO_2_ nanoparticles to develop intelligent packaging [67]. Red apple pomace is rich in anthocyanins and a percentage of 5% *w*/*w* was used to develop films, through the casting method. TiO_2_ was used to improve the stability and mechanical properties of the films [85]. Films were placed inside the top of a petri dish containing salmon and their colorimetric pH response was evaluated during 48 h. Films modified their color from red to a light pink following the pH variations from 6 to 8 that occurred when the extracts were exposed to different buffer solutions. The use in simultaneous of nanoparticles and anthocyanins not only improved the mechanical properties and resulted in a film with pH-sensing properties, but also enabled the films to possess a strong antioxidant and antibacterial capacity with future potential application in smart packaging.

Black rice, a source of anthocyanins, was used as a sensing material used in a matrix composed of oxidized-chitin nanocrystals and chitosan to develop films [69]. Films were prepared through the casting method and the anthocyanins extract was added in 0, 1, 3, and 5% *w*/*w* concentration. Films were placed on the surface of shrimp and promfet fish to evaluate its reaction as pH sensor. A color change was observed on the films with 3 and 5% *w*/*w* anthocyanins concentrations when exposed to food, which turned from an initial purple color to a grey–blue and brown, respectively. The total volatile basic nitrogen increased and was in solid accordance with the color changes observed, indicating food spoilage for both food samples. Furthermore, the films revealed a strong antioxidant capacity demonstrating a promising potential to be also applied as smart food packaging. 

It is worth mentioning that, despite most of the advances are based on the development of films with sensing properties providing a color change, other materials are being developed with the same purpose. Regarding this, a porous foam pH indicator using polyvinyl alcohol, polyvinyl pyrrolidone, and reinforced with microcrystalline cellulose was attempted using anthocyanins from red cabbage as sensing material [68]. In this work, a porous foam was developed using a freeze dryer and the resulting material was immersed on the anthocyanins extract. The resulting material was tested on prawns and chicken to evaluate its ability to work as a pH indicator. For both prawn and chicken, the indicator showed an initial purple color that faded into greenish–grey after 3 days of storage. The color changes followed the change of the pH environment that was initially acidic and became basic, showing the potential of the foams to be used in intelligent packaging as a colorimetric pH indicator for food spoilage. The assessment on antimicrobial and antioxidant activities was not done

Overall, anthocyanins are natural pigments, with antioxidant and antimicrobial properties and sensitivity to pH, so with interesting properties to construct a bio-based sensor for smart packaging systems [86,87,88]. Yet, it is of extreme importance to guarantee their stability to be used as indicators. Anthocyanins are highly sensitive to temperature alterations, which causes a loss of effectiveness of the product, so it is important to maintain their stability [89]. High temperatures leads to anthocyanins degradation, resulting in browning so lower temperatures are more desirable to maintain their stability [83]. Oxygen and light also contribute to anthocyanins degradation [58,89,90]. To overcome this constraint, bilayer films can be produced where the anthocyanins can be incorporated in the inner-layer, in contact with food, and compounds such as ZnO can be added to an outer layer of the matrix to counteract light induced degradation [58,89,90]. To develop a feasible film with sensing properties, the use of a proper biopolymer as the polymeric matrix for the incorporation of natural pigments is crucial [91]. The interactions of the biopolymer with the sensing material causes a rearrangement on the final matrix and causes structural changes of anthocyanins that influence the final color of the films [50]. The color of the films enriched with anthocyanins also depends on the nature of biopolymers, with a preference for neutral biopolymers such as starch and tara gum, that interacts with the pigment through hydrogen bonds thus with low interference on the color of the pigments [91,92]. In addition, the origin of the natural pigments is important to provide a visible color to the film, and to the color change promoted by the alterations of the food product, since anthocyanins from different origins have different colors and chemical structures [80,93]. Moreover, the concentration of anthocyanins incorporated is determinant to produce a successful film. An excessive amount can disrupt the structure of the film matrix caused by the aggregation of anthocyanins [91]. Regarding sensor-pigments, some studies indicate that it is important to immobilize them in the polymer, avoiding its migration to the food products, so that its sensor activity could be maintained. Yong and Liu suggests the addition of hydrophobic compounds to the film matrix to prevent undesirable migration to the food products [91]. Although pigments immobilization in the polymeric matrix will limit its activity, e.g., antioxidant or antimicrobial, still they can act as scavengers or absorbers or they can act directly to the area of the food that is in closed contact with the polymer. Additionally, mechanical and barrier properties of the films can be changed due to the incorporation of anthocyanins, and a balanced blend of reinforcements and plasticizers together with the pigments can help to maintain or enhance the food packaging efficiency [94]. The incorporation of anthocyanins in a biodegradable film is considered a promising alternative to indicators and sensors that are produced using non-degradable sources.

### 3.2. Betalains Used as Bio-Based Sensors

**Figure 3:**
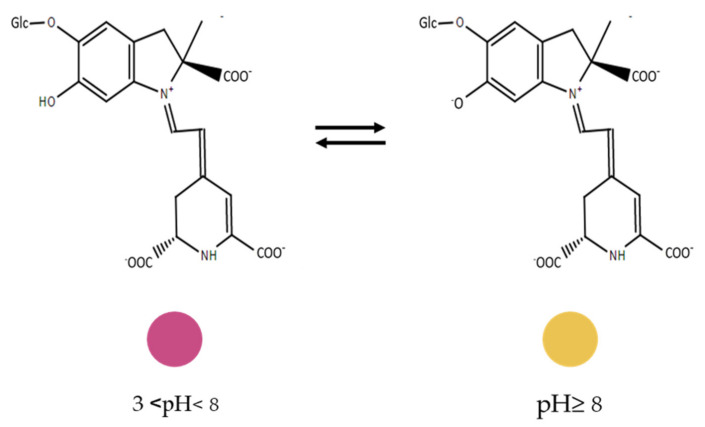
Changes in betalains structure due to pH changes, adapted from [3].

Betalains are water-soluble nitrogen-containing pigments located in the vacuoles of some plants tissue [95]. Betalains exhibit antioxidant capacity acting as radical scavengers, showing positive effects in the inhibition of lipid peroxidation and heme decomposition at a very low concentration [96,97]. Their common biosynthetic precursor is betalamic acid and these pigments appear to be stable between a pH from 3 to 8 (Figure 3) [95,97].

Yao and collaborators [70] developed quaternary ammonium chitosan and polyvinyl alcohol films (QAC-PVA) with the incorporation of betalains rich cactus pears extract (CPE) to act as a freshness indicator of fresh shrimp quality. Betalains are sensitive under alkaline conditions (pH ≥ 8), turning from red–violet to yellow–orange with the pH alterations (Figure 3). CPE was added to QAC-PVA blend and then dried. Films were introduced into the headspace of the shrimp container to evaluate its activity. The addition of CPE (2 and 3% *wt*%) provided color changes of the films, moving from an original purple color to an orange when the freshness of the shrimp decreased at 24 h [70]. Additionally, the addition of CPE to the films enhanced the UV-Vis light barrier, the elongation at break, antioxidant and antimicrobial properties. Films showed potential to be used in smart packaging due to their intelligent and active features, although its activity was only evaluated on the films and not on the food.

Another pH sensor for ammonia detection was reported, using amaranth as the source of betalains and a blend of quaternary ammonium chitosan and fish gelatin to form a film and evaluate the freshness of shrimp [71]. Betalains were extracted from amaranth and introduced in different concentrations (5, 10, and 15 *wt*%) in the filmogenic solution. The films were placed on the inner cover of the shrimp container and a color change from pink to yellow was observed, due to the betalains reaction to pH changes caused by the increase in volatile nitrogenous compounds. Moreover, the physical and functional properties of the films were studied demonstrating that the use of amaranth extract enhanced most of them. It was demonstrated that an increase in betalains concentration improved both the antioxidant and antimicrobial capacity of the films, however those activities were not accessed in situ. Those films demonstrated promising results to be used in smart packaging.

The same authors developed polyvinyl alcohol and starch films (2:1 starch:polyvinyl alcohol) incorporated with betalains extracted from red pitaya peel [72]. Betalains were extracted and incorporated into the film solution on different concentrations (0, 0.25, 0.50, 1 *wt*% starch basis). The inclusion of betalains extracts improved the film’s mechanical, water vapor, and light barrier properties when compared with the control films of pristine polymers. The films containing the betalains-rich extract were placed in the headspace of a petri dish containing fresh shrimps to test their freshness at 20 °C and for 48 h. Initially, the films possessed a pink color that was maintained for 24 h, but after, they turned into yellow, demonstrating its intelligent application. Moreover, the antioxidant and antimicrobial properties of the films were studied, indicating that the addition of this pigment improves those functionalities. Films with 1% of the betalain-rich extract were the most promising to be applied as a freshness indicator and due to their antioxidant and antimicrobial properties they have shown potential to be applied in smart packaging systems, which was not assessed in situ.

Kanatt and collaborators [73] developed active and intelligent films made of gelatin and polyvinyl alcohol with the incorporation of Amaranth leaf extracts mainly rich in betalains. The extracts were incorporated into the film-forming solution and films were prepared by the casting method. The addition of the extracts enhanced the physical and functional properties of the films. To be applied on fish and chicken, pouches were made through heat sealing, so in this case, the film was in contact with the food product. Films changed their color from red to yellow following the chicken and fish degradation. That color change was in accordance with the TVB-N content, pH, microbiological quality, and oxidative rancidity evaluated on the food products. Overall, the films demonstrated the potential to be used as smart packaging.

Betalains are gaining more attention due to their potential to be used as natural sensors in food packaging. Betalains stability is affected by extrinsic factors that include oxygen, temperature, and light [98]. To obtain the maximum potential as a colorimetric indicator it is important to maintain the functionality of the pigment. Pigment concentrations used to develop films must be carefully evaluated otherwise, they could affect the mechanical and physical properties of the films due to agglomeration of the pigments when used in excess [73]. Betalains when introduced in films tend to improve their physical, barrier and antioxidant properties [99]. Opacity is one of the parameters that are positively affected by the addition of these types of pigments by acting as a light barrier thus it may prevent food oxidative degradation [100,101]. Since betalains are more pH-stable than anthocyanins, their use in smart packaging constitutes a very promising alternative.

### 3.3. Curcumin Used as Bio-Based Sensor

**Figure 4:**
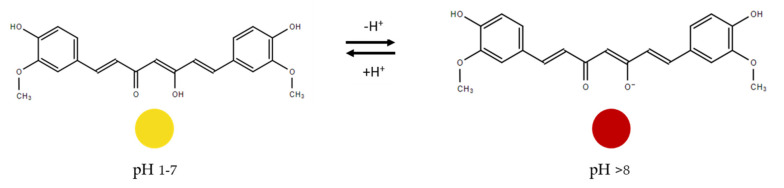
Changes in curcumin structure due to pH changes, adapted from [5].

Curcumin is a polyphenol extracted from the roots of turmeric and exhibits antioxidant, antimicrobial, anti-inflammatory activity and presents a strong yellow–orange color [44,49]. From a pH range of 1 to 7, it presents yellow coloration and above a pH of 8, curcumin color changes to red–orange [44] (Figure 4).

Curcumin was used as a sensing material to develop a film with tara gum and polyvinyl alcohol [77]. Different concentrations (0, 0.1, 0.3, and 0.5% *wt*%) of curcumin were added to the filmogenic dispersion, and films were developed through the casting method. Curcumin addition caused a slight decrease in the barrier properties of the films. To evaluate the sensing ability of the films to act as intelligent packaging, films were placed on the inside of a petri dish containing fresh shrimp, and the changes in color were studied at ambient temperature. At the initial time, films showed a yellow color and at the end of the experience presented an orange–red color. This was due to pH changes of the shrimps, caused by their degradation and resulting in the increment of the total basic volatile nitrogen content through ammonia production. The antioxidant and antimicrobial properties were not evaluated in this study. The film showed potential to be used in intelligent packaging.

In another study, agar-PVA films with curcumin provided a color change from yellow to orange–red when the shrimp freshness decline [76]. Hydrogel films were prepared by the casting method by dissolving the curcumin (0, 1, 2, and 3% *w*/*w*) on the agar and polyvinyl alcohol mixing solution. Additionally, a print label was developed through electrochemical writing. The ammonia sensitivity of the curcumin films was assessed using only the 2% *w*/*w* curcumin film, by placing it inside the shrimp container. In the presence of ammonia, pH increased and films could change their color, indicating shrimp degradation thus demonstrating potential to be used as an indicator in intelligent packaging. The antioxidant and antimicrobial properties were not accessed in this study. Moreover, the electrochemical writing on the films was accomplished adding value to the indicator, acting as a new possibility to provide information about the product.

Pectin-based films incorporated with curcumin and sulfur nanoparticles were developed to produce a film to monitor shrimp freshness during storage [78]. Films were prepared through the casting method. The addition of curcumin and sulfur nanoparticles improved thermal stability and UV-light barrier properties. Films were placed on the cover of the shrimp container and kept under 25 °C for 36 h to evaluate the colorimetric response to pH changes. Films changed from yellow to orange, caused by the changes on pH due to the production of volatile nitrogen compounds revealing shrimp degradation and, therefore, showing potential to be applied as intelligent packaging. Furthermore, films showed strong antibacterial and antioxidant properties to be applied as active packaging. However the effect of those parameters was not accessed directly on food products. This solution showed potential application in smart packaging.

Curcumin and *Lallemantia iberica* seed gum, as the matrix, were used to develop films to monitor shrimp freshness [79]. Films were prepared by the casting method using tween 80 as emulsifier. Films showed improvement in the water vapor barrier and also the mechanical properties when curcumin was added. Shrimps were covered with the films and stored at 25 °C and after 5 days, their color changed from an initial yellow to red color. These results followed the increase in TVB-N measured on the shrimps that lead to a pH change, causing the color changes. Moreover, the films containing curcumin revealed to have antimicrobial and antioxidant properties, thus demonstrating potential to be used also as active packaging. Yet, those effects were not tested directly on the preservation of food products. Overall, the film could be applied in smart packaging, either to monitor and to delay seafood degradation.

Curcumin possesses antimicrobial, and antioxidant activity, and it has the potential to participate as an active agent to extend certain food products shelf life [102]. When added to films their mechanical properties are not affected (at least with the quantities that have been studied) and its hydrophobic nature contributes to increase film’s hydrophobic properties [77]. In addition, the thermostability of curcumin may also improve the stability of the film [103]. The combination of its active properties with its indicator properties (color change with pH change) demonstrates clearly the great potential of the use of curcumin on films as a bio-based sensor for smart packaging.

Other natural pigments have been studied to be used as sensors in food packaging. Naphtoquinones are pigments that are sensitive to acid and basic environments so have the potential to be used as indicators [104,105]. Moreover, they can improve the moisture resistance of the films due to their hydrophobic character [106].

Naphthoquinone dye and cellulose extracted from *Arnebia euchroma* were used to develop a pH indicator for food spoilage [74]. Cellulose pulp was used to make a cellulose-based film, and naphthoquinone dye solution was introduced through impregnation on the film. Films were placed in the headspace of Petri dishes containing pork and shrimp samples. The increment of the total volatile basic nitrogen, as an important indicator for food spoilage [38], followed by a pH change, induced a colorimetric response of the films. Films demonstrated a response in a pH range from 5 to 12. A color change from rose–red (fresh) to purple (gentle spoilage) and finally to blue–violet (total spoilage) represented the different phases of food spoilage for both products. Neither antioxidant nor antimicrobial activities were evaluated on the films.

A similar pH sensor based on naphthoquinone (shikonin) and cellulose was also developed [75] and a similar method was applied as aforementioned to develop the color sensing film. Shikonin, a red naphthoquinone pigment, was extracted from gromwell root (*Lithospermum erythrorhyzon*) to be used as color sensor [75]. Film application on fish and pork packages showed a similar outcome, revealing a color change from red to blue as consequence of the pH increase in the environment due to the production of TVB-N. These authors also accessed the antioxidant activity of the films produced, revealing that shikonin enhanced this parameter on the paper demonstrating their potential to be used not only as intelligent packaging, but also as active packaging. In this regard, tests of that activity on food must be attempted to verify if there are beneficial effects on extending shelf-life. Moreover, both studies demonstrated that naphthoquinones are color stable under acidic conditions.

### 3.4. Other Potential Applications of Bio-Based Sensors

Although there is a lack of information about smart packaging systems for fruits and vegetables there have been some recent advances in the field. Fruits and vegetables are highly perishable, a consequence of the respiration process (CO_2_ release) and the ethylene production, which promotes faster degradation. Ethylene is a plant hormone that participates in the acceleration of the ripening of the fruits [107]. Scavenging and monitoring ethylene content, as well as reducing its amount in the packages atmosphere can provide valuable information and is important to avoid faster degradation of the perishables [108]. There are ripeness sensors commercially available as is the case of ripeSense^TM^, a sensor that reacts to aromas emitted by the ripening of the fruit, changing its color from an initial red to yellow [109]. The color changes indicate the fruits current ripeness level, allowing consumers to make a choice based on their preferences. A chemo resistive sensor was also developed to detect concentrations of ethylene at ppm levels using single-walled carbon nanotubes and a Cu(I) complex, that binds with the ethylene molecules, changing the resistance of the sensor which is then measured by the transducer [110]. More recently, a polydiacetylene-based sensor for ethylene detection was developed, based on a colorimetric response [111]. The sensor mechanism is based on the reaction between polydiacetylene and Lawesson reagent through the substitution of carboxylic groups with thiol, which when exposed to ethylene changed from blue to red following the increase in the ethylene concentration. Developing a bio-based sensor that could act in a similar manner would contribute to a more sustainable solution to pack fruits and vegetables.

Carbon dioxide (CO_2_) is an important food additive and food packaging quality indicator. When food starts to deteriorate, the CO_2_ levels start to increase mostly due to microorganisms growth [112]. Additionally, CO_2_ is used in modified atmosphere packaging to prevent microbial growth [49]. A CO_2_ indicator was developed based on the colorimetric response of a blend of lysine, ε-polylysine and anthocyanins [113]. This blend was introduced in an ethylcellulose solution to form a label and the effectiveness was assessed under simulated conditions and poultry meat. The use of ethylcellulose confers to the coating material a permeability to CO_2_ and impermeability to protons, avoiding pH-sensitive results. The CO_2_ detection by the sensor is directly caused by the alteration of the amino acid, and the formation of carbamic acid that causes a pH alteration and induces a colorimetric response by the anthocyanins [114]. The CO_2_ indicator label, under cold storage conditions, demonstrated to be reactive to CO_2_ exposure, changing from a blue to a purple color when the CO_2_ concentration increased, and demonstrating the potential to be used in monitoring food freshness. Oxygen is a disadvantageous trigger for food degradation, and in that sense, modified atmosphere packaging (MAP) is used to limit the interactions of the food products with the surrounding environment. Typically, it consists in low O_2_ concentration (0–2%) and high CO_2_ concentration (20–80%) [55]. Ageless Eye^TM^ is a commercially available redox indicator based on the color change of methylene blue to colorless when the dye is oxidized due to the increase in O_2_ levels when the package is compromised, acting as a leak indicator [115,116,117]. This type of solutions could be explored, modified, and tested in order to construct a bio-based sensor with potential to be applied in intelligent packaging with the same functionalities.

The use of nanosensors has a great interest in smart packaging due to their potential ability to detect, identify, and quantify pathogens, toxins, allergens, and other chemical markers [118]. When introduced in food packaging they can be useful to monitor food freshness and help in the determination of a more accurate shelf-life [119]. Several nanosensors have been developed and used in a wide range of applications, and a more detailed overview is given in lately published reviews [37,120,121]. However, and although some nanosensors have showed efficiency in detecting certain contaminants, their application in food packaging has not yet been applied.

Temperature is another determinant factor related to food spoilage that impacts food shelf life [55]. A critical temperature indicator based on a solvent melting point was developed, combining radio frequency identification with carbon nanotubes films [122]. This indicator is based on critical temperature. When this temperature is reached, the solvent flows through a capillary, soaks the carbon nanotube film and increases its resistance which is detected by the RFID. This type of sensor can be potentially useful to be used during storage through all the supply chain. A similar solution, more bio-based, could also be developed, in order to provide a structure that would be of interest by the food distribution chains, turning their business more sustainable.

## 4. Final Remarks and Future Trends

Food packaging is an important factor to preserve and maintain the quality of food products. The use of intelligent packaging constitutes an innovative path to provide more accurate information not only for the suppliers but most importantly for the consumers. Indeed, these new packaging systems, can be designed to inform the quality of the products being packed and allow to identify the degree of food deterioration, with benefits in terms of food safety and quality. Intelligent food packaging is also a promising area for the development of new and bio-based packaging systems fitting the actual global consumers’ demands and the awareness for the environmental issues caused by petroleum-based materials.

In this review, a focus was made on bio-based freshness indicators and their applications. These bio-based solutions provide information about the degree of freshness of a product and have been mostly employed in perishable products like meat, fish, and seafood. Most of the indicators tested used natural colorants and biopolymers (polysaccharides or proteins) and the freshness sensors developed were based on the pigments color change linked with a pH change. Once the components used can be obtained from biomass and biobased by-products, their development and application also contribute to a circular economy and a bioeconomy concept, in line with the Sustainable Development Goals of the United Nations and with the European Green Deal set by the European Commission.

The pigments anthocyanins, curcumin, and betalains, generally extracted from fruits and plants and their wastes, were the most studied options to develop the bio-based sensors. These pigments have also antimicrobial and antioxidant properties, and when integrated with a package they can also provide some activity to the biopolymer, helping to increase the shelf-life of the products being packed. Therefore, these bio-based sensors represent a potential application in smart food packaging systems. Despite their positive effect to be used as pH sensors, these pigments show some instability to temperature, O_2_, and light. So more studies are needed to improve the efficiency and stability of the indicators in the polymeric matrix. Encapsulation of the pigments can immobilize the compound inside solid particles or liquid vesicles to maintain stability, structure, and also to regulate the release of the pigment [89,99]. The use of nanoparticles, such as ZnO nanoparticles, as UV-light blocking agents is also an effective mechanism to protect and immobilize pigments [58,123]. Nanoclays, such as montmorillonite (MMT), commonly used with reinforcement purposes, also show promising results as pigments stabilizers [123,124]. Moreover, the combination of two different pigments to increase the sensor’s stability and the variety of color changes can be a novel strategy. For example, by mixing alcohol-soluble pigments and water-soluble pigments the possibilities to use as indicators are enhanced, becoming suitable for both water-soluble and fat-soluble foods [18]. The use of bilayers is also a strategy that can be applied to help stabilizing the pigments.

It would be also interesting to study a combination of these pigments with other active compounds (such as essential oils that can be obtained from a variety of aromatic plants or industrial crops), in the biocomposites, and to test them in situ, to certain types of food, to understand its potential as smart food packaging. Besides, there is a need to develop bio-based sensors that can be applied in fruits and vegetables, since these products are highly perishable and represent an economical loss and environmental problem. Additionally, bio-based packaging systems that can detect leaks in packages or that can detect pathogens, toxins, allergens, and other chemical markers, or that can detect how long a temperature breach has lasted across a cold chain, would definitely be welcomed by the food supply industries, once the amount of food waste produced could be reduced and the sustainability of the chain could be improved.

The use of biopolymers and natural pigments for intelligent and smart food packaging systems still shows some constraints and more studies are needed to better understand how the bio-based sensors interact with the food products. Even though most of the indicators developed do not contact directly with the food product, thus some migration and toxicological studies are needed.

Another key point is that, despite the innovation and development of new intelligent and smart packaging materials bio-based, there are still some limitations and, according to literature and market research, no suitable solutions are currently commercially available. One main reason is the increase in the cost of the packaging and the fact that existing solutions are not compatible with digital platforms. As most of the current bio-based sensors provide a visual response, it would be also interesting to develop systems that can provide a different response, e.g., information that can be emitted through a signal, readable, and transmitted through app notifications or LED (light-emitting diode) alarm messages. Therefore, highly sensitive, eco-friendly, low cost paper-based electrical sensors could be developed to spread the use of intelligent packaging [125]. This would contribute to minimize food waste and to provide value to by-products from lignocellulosic industrial crops such as giant reed or miscanthus [126,127].

## Data Availability

Data sharing not applicable.
